# Mutations in the DI-DII Linker of Human Parainfluenza Virus Type 3 Fusion Protein Result in Diminished Fusion Activity

**DOI:** 10.1371/journal.pone.0136474

**Published:** 2015-08-25

**Authors:** Wenyan Xie, Hongling Wen, Fulu Chu, Shaofeng Yan, Bin Lin, Wenli Xie, Ying Liu, Guijie Ren, Li Zhao, Yanyan Song, Chengxi Sun, Zhiyu Wang

**Affiliations:** 1 Department of Virology, School of Public Health, Shandong University, Jinan, China; 2 Department of Laboratory Medicine, Provincial Hospital affiliated to Shandong University, Jinan, China; 3 Department of Neurosurgery, Qilu Hospital of Shandong University, Jinan, China; 4 Shandong Center for Disease Control and Prevention, Jinan, China; 5 Department of Laboratory Medicine, Shandong Tumor Hospital and Institute, Jinan, China; 6 Institute of Biochemistry and Molecular Biology, School of Medicine, Shandong University, Jinan, China; 7 The Key Laboratory for Experimental Teratology of the Ministry of Education, Shandong University, Jinan, China; University of Minnesota, UNITED STATES

## Abstract

Human parainfluenza virus type 3 (HPIV3) can cause severe respiratory tract diseases in infants and young children, but no licensed vaccines or antiviral agents are currently available for treatment. Fusing the viral and target cell membranes is a prerequisite for its entry into host cells and is directly mediated by the fusion (F) protein. Although several domains of F are known to have important effects on regulating the membrane fusion activity, the roles of the DI-DII linker (residues 369–374) of the HPIV3 F protein in the fusogenicity still remains ill-defined. To facilitate our understanding of the role of this domain might play in F-induced cell-cell fusion, nine single mutations were engineered into this domain by site-directed mutagenesis. A vaccinia virus-T7 RNA polymerase transient expression system was employed to express the wild-type or mutated F proteins. These mutants were analyzed for membrane fusion activity, cell surface expression, and interaction between F and HN protein. Each of the mutated F proteins in this domain has a cell surface expression level similar to that of wild-type F. All of them resulted in a significant reduction in fusogenic activity in all steps of membrane fusion. Furthermore, all these fusion-deficient mutants reduced the amount of the HN-F complexes at the cell surface. Together, the results of our work suggest that this region has an important effect on the fusogenic activity of F.

## Introduction

Human parainfluenza virus type 3 (HPIV3) is an important pathogen of viral upper and lower respiratory tract illnesses such as bronchiolitis, croup, and pneumonia that require hospitalization all over the world in children aged younger than 5 years [1–4]. Currently, there are still virtually no effective preventive or therapeutic weapons available to help children to battle against it [5].

As a member of the paramyxovirus family, HPIV3 is an enveloped virus harboring a nonsegmented, negative-sense, single-stranded RNA genome. Fusion of the viral envelope with a host cell membrane is an essential step for its entry into the cytoplasm of the target cells to cause virus spread and the cell-cell fusion. It has evolved a membrane fusion mechanism that commonly involved two surface glycoproteins: the hemagglutinin-neuraminidase (HN) and the fusion (F) protein [6]. The HN protein is a type Ⅱ tetramer glycoprotein that carries out three discrete but critical functions (receptor recognition, neuraminidase and activating the F protein to induce membrane fusion) at specific points in the process of viral entry [7–9]. The F protein is thought to directly execute membrane fusion after its homologous HN protein binding to a host receptor [10].

**Fig 1:**
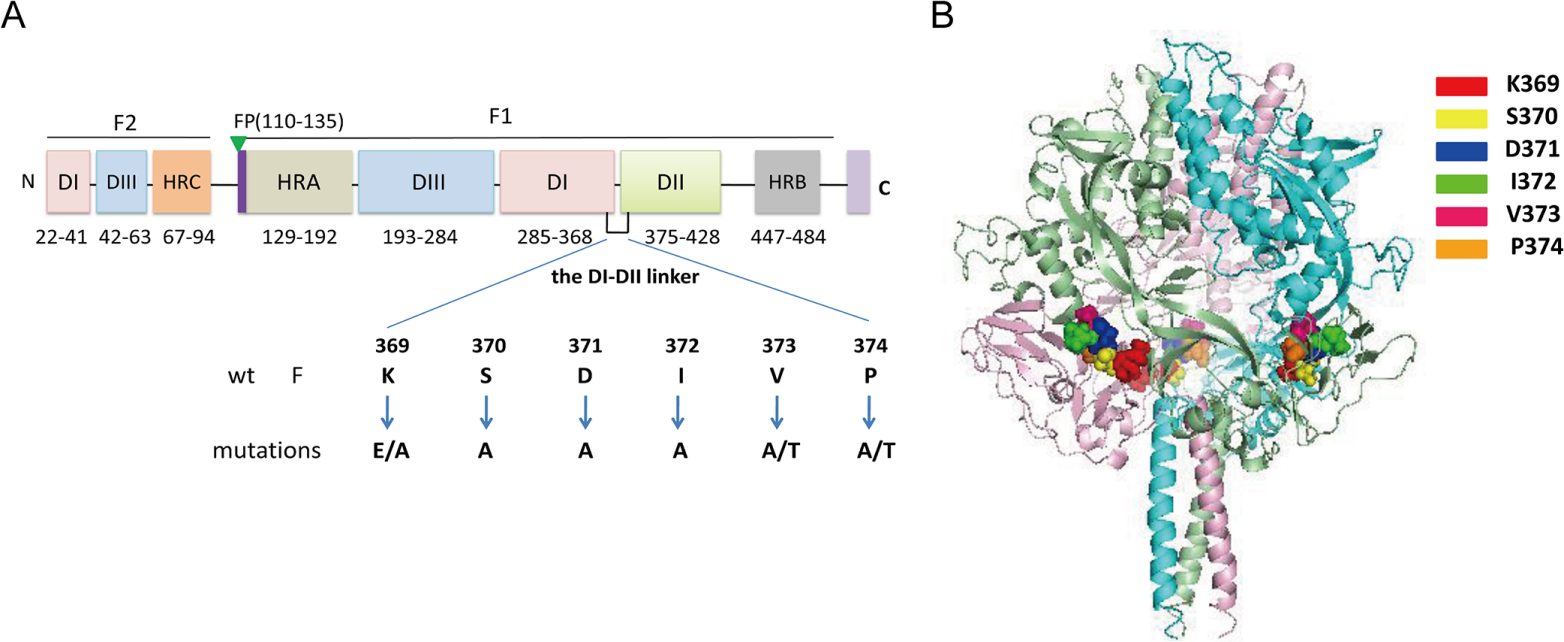
Schematic diagram of the locations of mutations. (A) Domain structure of the HPIV3 F protein and the relative location of the DI-DII linker in the primary sequence. This schematic is based on the secondary structure of the HPIV3 F protein in the postfusion conformation [16]. Structurally important domains are represented as different colors of rectangles, and their corresponding residue ranges are indicated. Individual mutations introduced into the region were listed beneath the wt sequence. Fusion peptide (FP), heptad repeat region (HR), transmembrane domain (TM), cytoplasmic tail domain (CT), and structural domains DI, DII, and DIII are shown. (B) Locations of the DI-DII linker (residues 369–374) in the prefusion HPIV3 F protein structural model. A side view of the ribbon diagram of the structural model of the HPIV3 F protein in the prefusion conformation which was generated based on the crystal structure of the prefusion PIV5 F protein (PDB ID 4GIP) is shown. The three monomers are shown in light pink, aquamarine, and pale green. The mutant residues are shown in space-filling mode in three chains. The figure was generated with PyMOL 0.99.

The HPIV3 fusion (F) glycoprotein, belonging to the class Ⅰ virus membrane fusion protein [6], is synthesized as an inactive precursor (F0) which is cleaved by a host cell proteolytic enzyme in the trans Golgi apparatus into a biologically active form consisting of a larger carboxy-terminal subunit (F1) and a smaller amino-terminal subunit (F2) [11]. The F1 subunit contains the fusion peptide (FP), which is thought to make the primary contact with the target cell membrane upon the onset of fusion [6, 12], the transmembrane (TM) domain which is needed for fusion pore formation [13], and two hydrophobic heptad repeat regions designated HRA and HRB, with HRA adjacent to the FP and HRB proximal to the TM. In the process of membrane fusion, HRB is known to pack along the outside of the trimeric HRA coiled coil to form a conserved six-helical bundle (6HB) that pulls the viral and target cell membranes together [6, 14, 15]. Furthermore, between the two heptad repeat domains there are about 250 amino acid residues which comprise three domains (DI-DIII) and two linkers (the DI-DII linker and the HRB linker). It is noteworthy that only the X-ray crystallographic structure of the HPIV3 F protein in the postfusion conformation is currently available [16] and HRA, HRB, domains DI-DIII and the two linkers have been found in this structure (Fig 1A). Up to now, although several F protein regions have been considered to have important effects on regulating the membrane fusion activity, whether the DI-DII linker of the HPIV3 F protein has an effect on fusogenicity is as yet unclear.

To explore the role of this domain in fusion activity, the homology structural model of the HPIV3 F protein in the prefusion conformation based on the PIV5 prefusion F-trimer structure (PDB ID 4GIP) was initially generated to locate the new position of this linker in it, then site-directed mutagenesis was employed to substitute each amino acid residue in this domain to form K369A, K369E, S370A, D371A, I372A, V373A, V373T, P374A, P374T mutants. A transient expression system using the vaccinia virus-T7 RNA polymerase was used to express the wild-type or mutated F proteins [17]. The effects on fusogenicity, cell surface expression, and interactions between F and HN proteins of these mutated F proteins were examined. The results showed that all mutant proteins in this domain which still acted as the DI-DII linker in the prefusion form detected at the cell surface were present at approximately wild-type levels, but they were deficient in fusion activity in all steps of the fusion process when coexpressed with their homologous HN proteins. The loss of fusogenic activity correlates with the decreased ability of the mutated F proteins to interact with the homologous HN protein at the cell surface.

## Materials and Methods

### Homology Modeling

A homology modeling of the HPIV3 F protein in the prefusion conformation was generated on the basis of the crystal structure of the prefusion PIV5 F protein (PDB ID 4GIP) on the SWISS-MODEL protein-modeling server [18, 19]. Each monomer was individually built and then merged into a trimer, which was the most likely prefusion structure of the HPIV3 F.

### Cells and viruses

BHK-21 cells, obtained from the American Type Culture Collection, were propagated in Dulbecco’s modified Eagle’s medium (DMEM) (Gibco) supplemented with 1% glutamine, 10% fetal calf serum (FCS) (Gibco), and 1% penicillin-streptomycin (Invitrogen).

Wild-type (wt) vaccinia virus was used to quantify cell fusion, while recombinant vaccinia virus vTF7-3, a generous gift from Dr. Bernard Moss, was maintained in BHK-21 cells to provide T7 RNA polymerase in the vaccinia-T7 RNA polymerase expression system [17].

Fresh human erythrocytes (RBCs) were prepared from physical examination center of Qilu Hospital of Shandong University. This study was approved by the Institutional Review Board of Shandong University and the hospital ethics committee. Written informed consent was obtained from all enrolled patients.

### Recombinant plasmids vectors and transient expression system

The recombinant plasmid vectors were donated by Professor Ronald M.Iorio. HPIV3 HN and F genes were inserted into pBluescript SK(+) (pBSK^+^) at the BamHⅠ site, respectively, to generate pBSK-HN and pBSK-F [20]. The recombinant plasmids were obtained from transformed *Escherichia coli* TG1 cells and were purified using ENZA Plasmid Miniprep Kit (Omega Bio-Tek, Inc., USA).

The vaccinia virus-T7 (vTF7-3) RNA polymerase expression system was used to express the wild-type (wt) and all mutated F proteins. BHK-21 cells were seeded in six-well plates or on 60-mm-diameter tissue culture plates at 4×10^5^ cells per well a day prior to transfection. Confluent monolayers of cells were infected with recombinant vaccinia virus at a multiplicity of infection (MOI) of 10 PFU. The virus was adsorbed for 1 h at 37°C in a 5% CO_2_ incubator and then washed with serum-free DMEM. As recommended by Dutch et al [21], cells were then transfected with Lipofectamine 2000 (Invitrogen Biotechnology) and 1 μg of each plasmid.

### Site-directed mutagenesis

Site-directed mutagenesis was utilized according to protocols previously described [20], using HPIV3 F recombinant plasmid vector as the template in each PCR mutagenesis to generate the target amino acid substitution. Two pairs of reverse-complement mutagenesis primers (Sangon Biotech Co. Ltd., Shanghai, China) were also used. The primers are as follows (sequences were started with the 5' nucleotide and in all primer sequences altered nucleotides were underlined):

VP1 (vector): 5'-GTG ACT GGT GAG TAC TCA ACC AAG TC -3'

VP2 (vector): 5'-GA CTT GGT TGA GTA CTC ACC AGT CAC -3'

K369A-P1: 5'-AGA ACC GTG GTT GCA TCA GAC ATT G-3'

K369A-cP1: 5'-C AAT GTC TGA TGC AAC CAC GGT TCT-3'

K369E-P1: 5'-A ACC GTG GTT GAA TCA GAC ATT GTT-3'

K369E-cP1: 5'-AAC AAT GTC TGA TTC AAC CAC GGT T-3'

S370A-P1: 5'-ACC GTG GTT AAA GCA GAC ATT GTT C-3'

S370A-cP1: 5'-G AAC AAT GTC TGC TTT AAC CAC GGT -3'

D371A-P1: 5'-TG GTT AAA TCA GCA ATT GTT CCA AG -3'

D371A-cP1: 5'-CT TGG AAC AAT TGC TGA TTT AAC CA -3'

I372A- P1: 5'-GTT AAA TCA GAC GCT GTT CCA AGA T -3'

I372A-cP1: 5'-A TCT TGG AAC AGC GTC TGA TTT AAC -3'

V373A-P1: 5'-A TCA GAC ATT GCT CCA AGA TAT GCA -3'

V373A-cP1: 5'-TGC ATA TCT TGG AGC AAT GTC TGA T -3'

V373T-P1: 5'-A TCA GAC ATT ACT CCA AGA TAT GCA-3'

V373T-cP1: 5'-TGC ATA TCT TGG AGT AAT GTC TGA T-3'

P374A-P1: 5'-TCA GAC ATT GTT GCA AGA TAT GCA T -3'

P374A-cP1: 5'-A TGC ATA TCT TGC AAC AAT GTC TGA -3'

P374T-P1: 5'-TCA GAC ATT GTT ACC AGA TAT GCA T-3'

P374T-cP1: 5'-A TGC ATA TCT GGT AAC AAT GTC TGA-3'

Briefly, the pBSK-F plasmid digested by KpnⅠ was used as the template to generate one PCR fragment with primers K369A-P1 (complemented with K369A-cP1) and VP2 (complemented with VP1). The pBSK-F plasmid digested by NotⅠ was used as the template to create the other PCR fragment with primers K369A-cP1 (complemented with K369A-P1) and VP1 (complemented with VP2). Two PCR products with the short homologous sequene in the products terminal were transformed into *Escherichia coli* TG1 cells, then the transformed cells were selected for ampicillin resistance. Two unique restriction sites (KpnⅠ site and NotⅠ site) were used to facilitate screening the colonies carrying mutated F genes. Finally, DNA sequencing was performed to verify that the desired mutation(s) had been successfully introduced. According to the above procedures, we obtained the other single mutants successfully.

### Syncytium formation assay

BHK-21 cells were grown in 6-well plates a day earlier before transfection. At ~ 80% confluence, the cells were cotransfected with wt F or different mutant F genes along with their homologous wt HN genes or empty vector alone. At 36 h post-transfection, monolayers were washed with phosphate-buffered saline (PBS) after removal of the complete medium, fixed with methanol for 5 min and stained with Giemsa Accustain (Sigma Chemical Co) for syncytium observation [22]. An inverted microscope (Olympus IX71) was used to capture representative fields. In each field of view, all syncytia with three or more nuclei were counted. Quantification of syncytia was completed by measuring the area covered by syncytia in Adobe Photoshop CS6, referring it to the total area of the field for three random fields [23].

### Content mixing assay

A modification of the reporter gene assay was performed to quantify the ability of F to mediate cell-cell fusion as previously described [24, 25]. Briefly, monolayers of BHK-21 effector cells already infected with the recombinant vaccinia virus 1 h earlier at 37°C were cotransfected with the desired F and their homologous HN genes. Monolayers of BHK-21 target cells already infected with wild-type vaccinia virus at a MOI of 10 1 h before at 37°C were transfected with 1 μg of plasmid pG1NT7β-gal which encodes β-galactosidase. Following a 22 h incubation at 37°C, the cells were removed from the wells with 0.05% trypsin and 0.53 mM EDTA (Gibco), washed with DMEM, and resuspended in complete medium.

Equal numbers (1×10^5^) of the effector and target cells were combined in duplicate wells of a 96-well microtiter plate. After 5 h incubation at 37°C, the cells were lysed and the extracts were assayed for β-galactosidase activity according to the procedures of High Sensitivity β-Galactosidase Assay Kit (Stratagene, La Jolla, CA, USA). The level of fusion was quantified by measuring the absorbance at 590 nm with a plate reader (ELX800; Bio-tek Instruments, Inc., Winooski, Vt.) by subtracting background fusion of BHK-21 cells transfected with comparable amounts of the vector alone [26].

### Dye-transfer assay

RBCs were washed, resuspended in cold PBS (1% hematocrit), and then incubated with 15 μl octadecyl rhodamine B (R18, Invitrogen) (1 mg/ml in ethanol). Unbound probe was absorbed by the addition of complete medium. The labeled RBCs were then washed and resuspended in cold PBS containing 0.1 mM CaCl_2_ and 1 mM MgCl_2_ (PBS-CM) (0.1% hematocrit) for further use. At 22 h post-transfection, cell monolayers coexpressing the wt or mutated F and their HN proteins were washed and incubated with 50 mU per ml of neuraminidase (Sigma Chemical Co.) at 37°C for 1 h, and then washed again. The R18-labeled RBCs were added and incubated at 4°C for 30 min. Cells were washed, then incubated at 37°C for 1h, and a Nikon fluorescence microscope was used to count and photograph the events of dye transfer [27].

### The kinetics of fluorescence dequenching assay

The kinetics of dequenching of fluorescence R18 was measured as previously described [21, 28]. RBCs were labeled with R18 in the same way as in the dye-transfer assay. BHK-21 cells were seeded on 6-cm-diameter dishes containing a coverslip (~80% confluence) and infected with vTF7-3 at a MOI of 10 PFU per well for 1 h. Then the cells were transfected with the same ratio of wt or mutated F and HN DNAs (2.5 μg: 2.5 μg), with the total DNA transfected for each dish kept at 5 μg. After 22 h incubation, transfected cells were washed and incubated with neuraminidase (Sigma Chemical Co.) as described above. After being washed twice, cells were incubated with R18-labeled RBCs (0.1% hematocrit) at 4°C for 30 min. Monolayers were washed and the RBC-cell complexes were removed from the dish into a new 2.0 ml Eppendorf tube using 50 mM EDTA in PBS at 4°C. After being washed, the complexes were placed on ice for the downstream experiment. A 50μl volume of R18-labeled RBC-acceptor cell suspension was injected into a cuvette containing 3 ml of PBS prewarmed to 37°C, and a Hitachi F-4500 spectrofluorometer (Japan) (excitation wavelength, 560nm; emission wavelength, 590nm) was used to monitor the changes of fluorescence continuously with a 5 s time resolution. The percent fluorescence at any time was calculated as 100(F - F_0_ / F_t_ - F_0_), where F_0_ is the fluorescence intensity at time zero, F is the fluorescence intensity at a given time point, and F_t_ is the fluorescence intensity after the addition of 0.1% Triton X-100, which was considered to result in infinite dilution of the probe [29].

### Flow cytometry

Fluorescence-activated cell sorter (FACS) analysis was performed to assay the cell surface expression of the HPIV3 wt and each mutated F proteins as previously described [22]. At 22 h posttransfection, the monolayers were removed from plates by treatment with 5 mM EDTA in PBS, pelleted by centrifugation, washed twice, and then incubated with a monoclonal antibody (CD9-4-3; Millipore) specific for the HPIV3 F protein at a dilution of 1:100 for 1 h at room temperature. Then the cells were washed and incubated with 1:100 diluted goat anti-mouse immunoglobulin G coupled to Alexa Fluor 488 dye (Proteintech) for 1 h at room temperature. After being washed, the cells were fixed in PBS containing 2% paraformaldehyde at 4°C for 10 min. Following two more washes, cells were resuspended in 0.4 ml of PBS for analysis in a FACS Calibur flow cytometer (Becton Dickinson Biosciences). Cells transfected with vector alone as controls were also incubated with both primary and secondary antibodies.

### Co-immunoprecipitation assay

The abilities of HPIV3 wt or mutated F proteins to interact with their homologous HN proteins were assayed by using a modification of co-immunoprecipitation assay [20, 30, 31]. Monolayers of BHK-21 cells were seeded in 6-well plates (~80% confluence) and transfected with HPIV3 wt or mutated F genes together with HN genes as described above. At 22 h post-transfection, cells were washed and incubated with membrane-impermeable EZ-Link sulfo-NHS-SS-biotin (Pierce, Rockford, Illinois, USA.) for 30 min at 4°C with mild rocking. Excess reagent was removed and the cells were lysed. The lysates were clarified by centrifugation and the supernatants were collected into a 1.5 ml Eppendorf tube which contained 50 μl anti-HN antibody at a 1:100 dilution that had been cross-linked to Dynabeads protein A (Invitrogen). The anti-HN antibody purchased from Abcam contains two monoclonal antibodies (M02122321 and M0301307). The mixes were incubated with rotation in order to form Dynabeads-HN Ab-HN Ag-F Ag complexes. Then the complexes were washed with a magnet, boiled, re-suspended in PBS and incubated with streptavidin beads (Pierce) all night at 4°C. After being washed, the samples were re-suspended in gel sample buffer.

### Polyacrylamide gel electrophoresis and Western blot analysis

Proteins diluted in gel sample buffer with 1 mol β-mercaptoethanol, were separated in 10% polyacrylamide gels as previously described [31]. After electrophoresis, gels were equilibrated in transfer buffer and transferred to Immobilon-P (Millipore Corp.) membranes. The membranes were blocked, washed with PBS-Tween 20, and incubated with primary antibody (either a MAb specific for the F protein or a mixture of two anti-HN MAbs) at a 1:100 dilution in PBS-Tween 20 and 0.5% nonfat milk overnight at 4°C. The anti-F antibody (MAB10207) is a mouse monoclonal antibody and is purchased from Chemicon. After being washed, membranes were incubated with goat anti-mouse immunoglobulin G coupled to horseradish peroxidase (ZSGB-BIO, Beijing, China) diluted to 1: 2,000 in PBS-Tween 20 for 1 h. Following extensive washes, protein bands were visualized by enhanced chemiluminescence (ECL; millipore) and detected using an ECL detection system (Amersham Biosciences). The quantification of the signal was accomplished using a Fluor-S imager (Bio-Rad).

### Statistical analysis

All results were expressed as the mean ± SD from at least three separate experiments. Statistical analysis was conducted using Student’s t-test, *P* < 0.05 was considered significant.

## Results

### Mutagenesis of each amino acid residue in the DI-DII linker of the HPIV3 F protein

As shown in Fig 1A, the DI-DII linker is located between domains DI and DII at residues 369 to 374 in the HPIV3 F protein sequence. Fig 1B shows the localization of this linker (residues 369–374) in the 3-D structure of the HPIV3 F protein in the prefusogenic conformation. This homology model was generated based on the PIV5 prefusion F-trimer structure (PDB ID 4GIP). The reason why we firstly reposition the residues 369–374 in the prefusion structure of HPIV3 F is that the postfusion F protein is no longer fusogenic. Interestingly, the residues 369–374 correspond to the residues of DI-DII linker of the PIV5 F protein by amino acid sequence alignment. Therefore, the DI-DII linker in the postfusion structure of HPIV3 F still served on the DI-DII linker in its prefusion form. With the goal of probing the effect of this domain on fusion activity, each amino acid residue in this domain was substituted to form the following mutants: K369A, K369E, S370A, D371A, I372A, V373A, V373T, P374A, P374T (Fig 1A). Alanine was introduced because it has minimal disruptive impact on protein structure.

### Amino acid substitutions in the DI-DII linker exhibited diminished fusion activity in all steps of the fusion process when coexpressed with their homologous HN proteins

To explore the effects of these single amino acid substitutions in the DI-DII linker of the F protein on the fusogenic activity, three different types of membrane fusion assays were employed.

**Fig 2:**
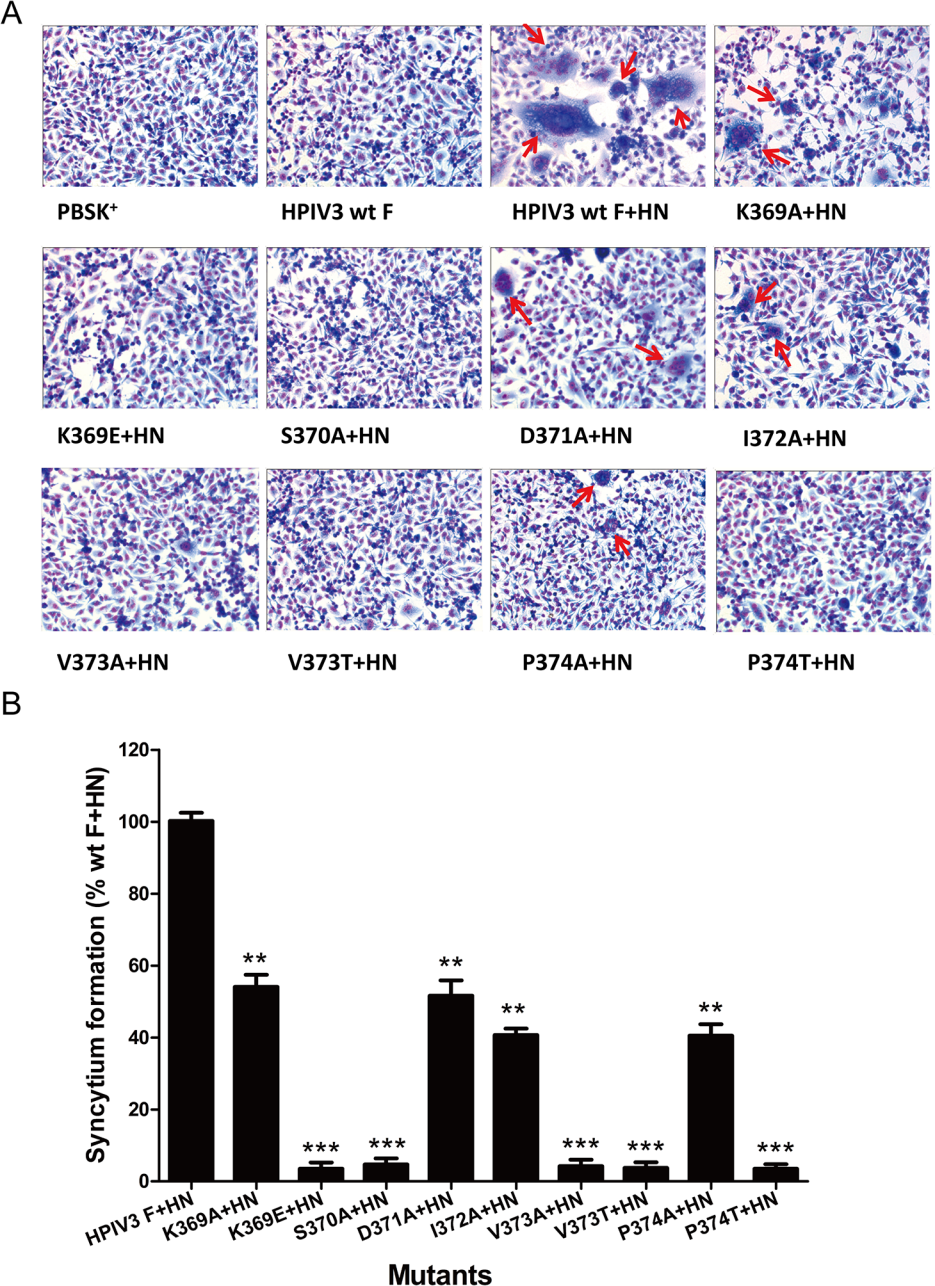
Syncytium formation in monolayers coexpressing wt or mutated F and HN proteins. After 36 h posttransfection, BHK-21 monolayers transfected with vector alone, wt F alone, wt F and wt HN, or mutant F and wt HN were fixed with methanol and stained with Giemsa stain. (A) Photomicrographs from a representative expreriment. Red arrows indicate syncytia. (B) Quantification of syncytia produced by the mutated F proteins. Values were indicated as percentages of syncytium formation detected in cells transfected with wt F and wt HN proteins and are represented as the mean ± standard deviation (SD) from three separate experiments (*P<0.05, **P<0.01, ***P<0.001).

We initially measured the extent of syncytium formation to evaluate an overall level of cell-cell fusion. HPIV3 wt or mutated F proteins were coexpressed in BHK-21 cells with their homologous HN proteins. After 36 h posttransfection, an inverted microscope was used to observe multinucleated giant cells and representative photomicrographs of syncytia were shown in Fig 2A. The cells expressing HPIV3 wt F protein or vector alone were used as negative controls. Under the microscope, the syncytia formed by these mutants in the presence of their homologous HN protein coexpression were not only smaller but also fewer in comparison to those produced by wt F and HN proteins. Quantification of syncytia, expressed as a percentage of those detected in cells coexpressing wt F and HN proteins, is shown in Fig 2B. When coexpressed with HN, all mutant F proteins displayed decreased levels of syncytium formation as compared with the HPIV3 wt F and HN proteins. The F proteins carrying individual K369E, S370A, V373A, V373T and P374T mutations almost abrogated their abilities to form syncytia, with extents less than 5.0% of the wt F and HN level. The remainder of the mutants in this domain (K369A, D371A, I372A and P374A) were capable of forming syncytia, though at lower levels than seen with the wt F protein.

**Fig 3:**
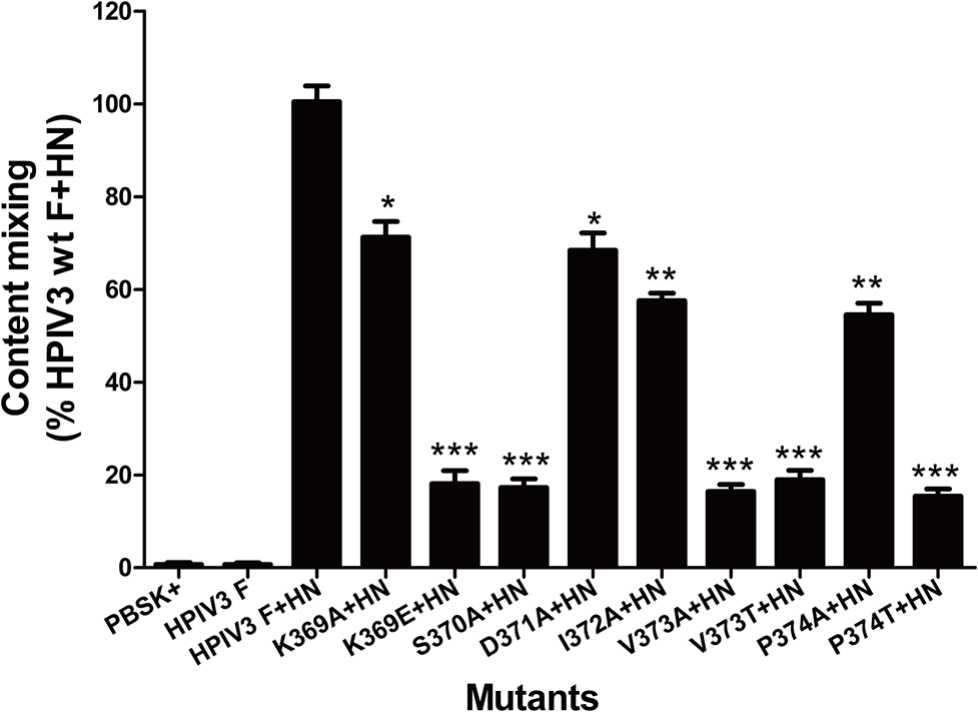
Content mixing of wt and mutant F proteins. Quantification of content mixing directed by wt or mutant F proteins with their homologous HN proteins as measured by β-galactosidase activities. Values are expressed as percentages of content mixing detected in cells transfected with wt F and HN proteins. The experiment was performed in triplicate; the data are shown as the mean±SD (*P<0.05, **P<0.01, ***P<0.001).

To more accurately measure the fusogenicity of the F protein regulated by these mutations, a modification of the reporter gene assay was carried out as detailed in Materials and Methods to measure content mixing. Two populations of cells are indispensable for this assay: BHK-21 effector cells and BHK-21 target cells. Values are expressed as percentages of content mixing detected in cells transfected with wt F and HN proteins. The data summarized in Fig 3 showed that five mutations (i.e., K369E, S370A, V373A, V373T and P374T) that almost eliminated their abilities to form syncytia also resulted in debilitated content mixing, which retained only 18.0%, 17.3%, 16.4%, 18.9%, 15.4% of the wt F level, respectively, when coexpressed with their homologous HN proteins. While the K369A and D371A mutants directed approximately two-thirds of the levels of content mixing of the wild-type protein, the I372A and P374A mutants reduced the levels of content mixing to 57.6% and 54.5% of that of wt F, respectively. Therefore, all the mutated F proteins were defective to various degrees in content mixing.

**Fig 4:**
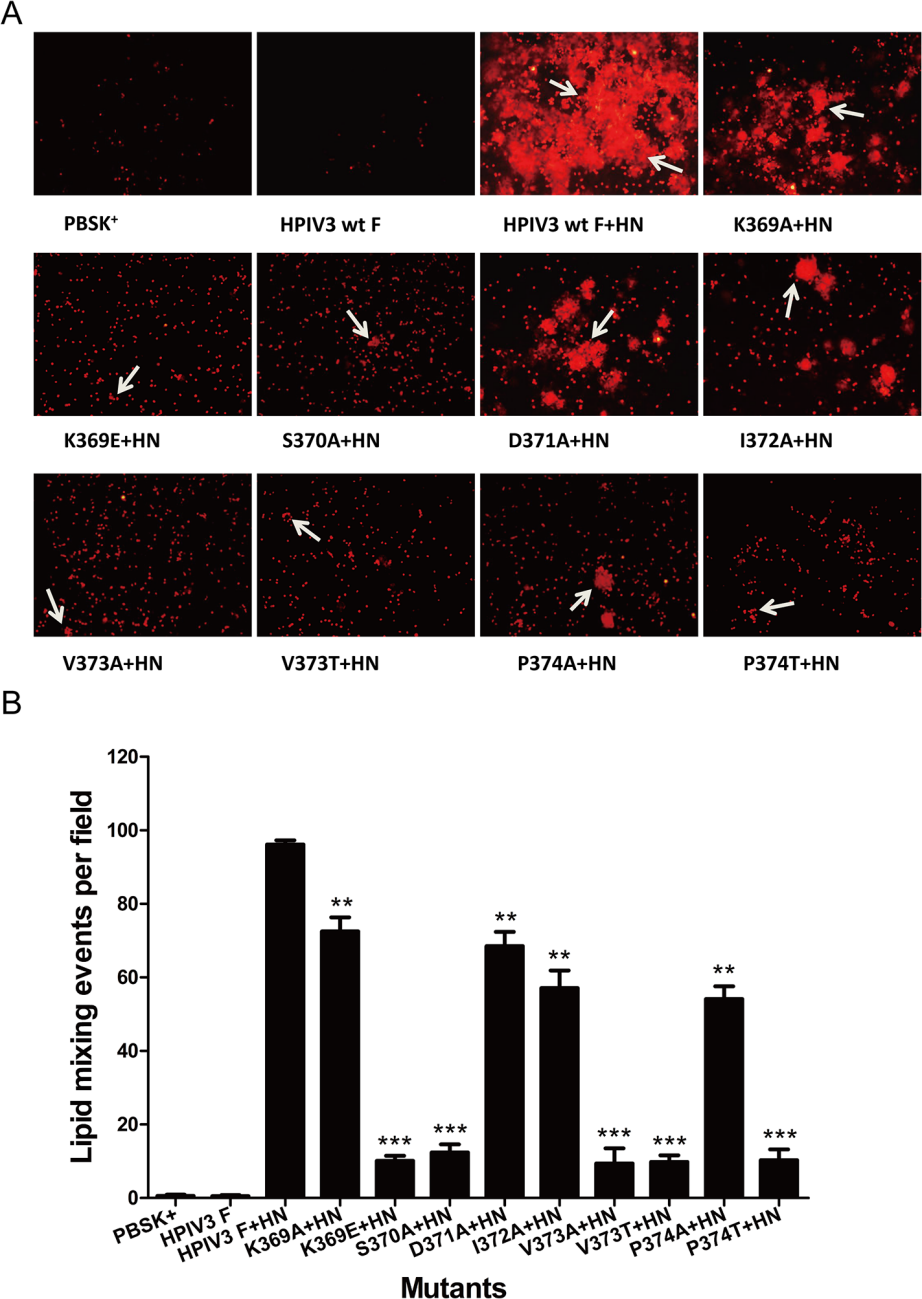
Dye transfer directed by the wt or mutated F and HPIV3 HN proteins. R18-labeled RBCs were bound at 4°C to BHK-21 cells coexpressing the wt or mutanted F and HN proteins. Cells were incubated for 60 min at 37°C to allow membrane fusion, images were acquired by using fluorescent microscopy. (A) Representative images of dye transfer. White arrows indicate the lipid mixing events. (B) Quantification of the events of dye transfer. The extent of dye tranfer is expressed as the average number of R18 lipid dye tranfer events of six microscopic fields. The means and standard errors are from six microscopic fields (**P<0.01, ***P<0.001).

Membrane fusion is usually divided into three successive phases [32, 33]: hemifusion, pore formation and pore enlargement. To address whether the mutated F proteins that are deficient in pore formation and pore enlargement are capable of mediating hemifusion, we carried out a fluorescent dye transfer assay with R18-labeled RBCs to assess the extent of the lipophilic probe R18 transfer from RBC membranes to transfected BHK-21 cell membranes, which occurs during the initial stages of membrane fusion. Fig 4A shows the representative photomicrographs of the dye-tranfer assay performed with cells coexpressing HPIV3 wt or mutated F and HN proteins. The controls for this experiment showed that there is no dye transfer from the labeled RBCs into cells expressing pBSK^**+**^ or wt F protein alone, while dye transfer can be observed from the labeled RBCs into underlying cells coexpressing wt or mutant F and HN proteins. The extents of dye transfer were represented as the average number of dye transfer events of six microscopic fields. It can be seen from Fig 4B that in the presence of HPIV3 HN coexpression, the proteins carrying K369E, S370A, V373A, V373T and P374T substitutions reduced the extents of dye transfer drastically, corresponding to a percentage of less than 12.5% of that of the wt F. The remaining mutated F proteins (i.e., K369A, D371A, I372A and P374A) dropped the extents of dye transfer to 72.5%, 68.5%, 57.1% and 54.1% of wt F level, respectively. Therefore, mutations in the DI-DII linker restrained membrane fusion in the earliest phase.

**Fig 5:**
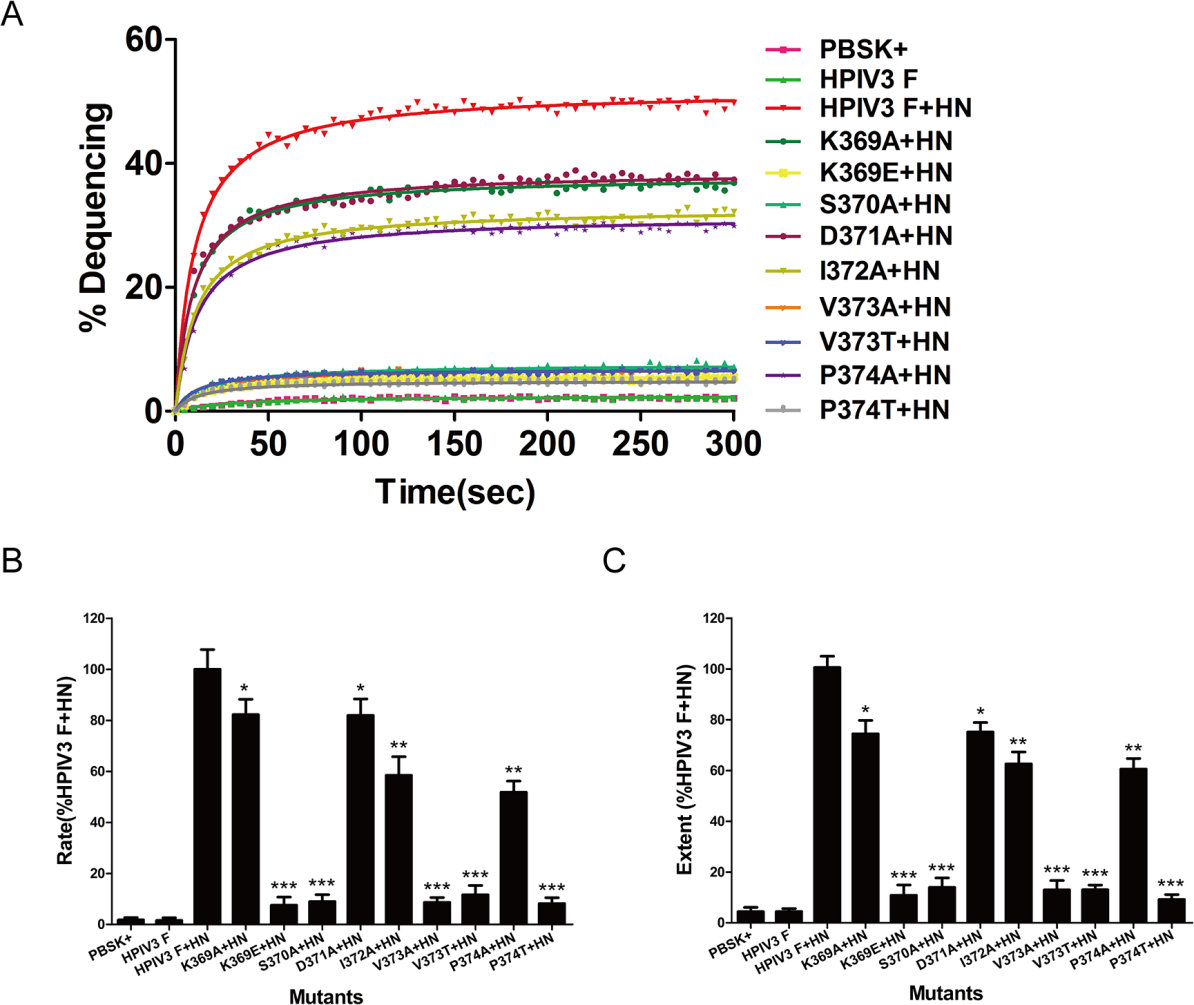
Kinetics of R18 dye transfer for HPIV3 wt or mutated F and HN proteins. The effector-target cell complexes was injected into a cuvette containing PBS prewarmed to 37°C, and fluorescence changes were measured as described in Materials and Methods. (A) The kinetics of dye transfer from R18-labeled RBCs to effector cells. A single point that represents data obtained from three different experiments. GraphPad Prism 5.0. was used to perform nonlinear regression on data points. (B) Determination of the initial rates of fusion of the target-effector cell complexes. The rates of hemifusion induced by the mutated F and HN proteins were calculated from maximum slopes of fit curves as shown in Fig 5A, and normalized to the maximum rate of hemifusion induced by wt F and HN proteins. Data are means±SD of three independent experiments (*P<0.05, **P<0.01, ***P<0.001). (C) Determination of the maximum extent of hemifusion. The maximum extent of fluorescence dequenching at 5 min was calculated from kinetics curves analogous to those shown in Fig 5A. The data are expressed as the percent activity relative to the wt F and HN level. The results are the mean±SD (*P<0.05, **P<0.01, ***P<0.001) from three separate experiments.

In order to acquire further quantitative information concerning the rate and extent of hemifusion induced by wt or mutant F proteins in the presence of HPIV3 HN protein coexpression, the kinetics of fluorescence dequenching of the lipophilic R18 probe was monitored. The R18 probe is readily inserted into RBC membranes that were then mixed with unlabeled BHK-21 acceptor cells. Fusion of R18-labeled RBCs with the acceptor cells coexpressing the HPIV3 wt or mutated F and HN glycoproteins led to the dispersion of the probe R18, that is to say, the fusion resulted in increased excimer fluorescence which can be monitored by a spectrofluorimeter. As shown in Fig 5A, no fluorescence dequenching was detected when cells individually expressing HPIV3 F protein or vector were incubated with R18-labeled RBCs in this assay (hotpink curve or green curve). However, fluorescence dequenching increased rapidly and significantly (red curve) when R18-labeled RBCs were mixed with cells coexpressing the wt F and HN proteins. The initial rates were calculated from the maximum slopes of curves as illustrated in Fig 5A (Fig 5B), and the values are normalized to the maximum rate of hemifusion mediated by wt F and HN proteins. The maximum extent of hemifusion at 5 min were calculated from kinetics curves as exemplified in Fig 5A (Fig 5C), and the data are represented as the percent activity relative to the wt F and HN level. Only very little fluorescence dequenching was detected between cells coexpressing K369E, S370A, V373A, V373T or P374T mutated F proteins along with their homologous HN proteins and R18-labeled RBCs, both the rate and extent of the initial fusion were less than 15% of those of the levels of wt F and HN. The extents and rates of hemifusion of K369A and D371A mutated F proteins coexpressed with HPIV3 HN protein were about three quarters (74.5%, 75.2%, respectively) and four fifths (82.2%, 82.0%, respectively) of those for the wt F and HN. In addition, the I372A and P374A mutants in the presence of HPIV3 HN protein coexpression decreased the initial rate to 58.5% and 51.8%, respectively, and diminished the initial extent to 62.6% and 60.6%, respectively. Thus, mutations in the DI-DII linker of HPIV3 F protein weakened the rates and extents of hemifusion. The qualitative and quantitative results obtained from the dye-transfer assay and the kinetics of fluorescence dequenching assay are coincident with those obtained from both the syncytium formation assay and the content mixing assay. Taken together, when the HPIV3 F proteins with amino acid substitutions in the DI-DII linker were coexpressed with their homologous HN proteins, each of them exhibited a significant deficiency in fusion activity in all steps in fusion.

### Substitutions in the DI-DII linker had no effect on the cell surface expression of F

**Fig 6:**
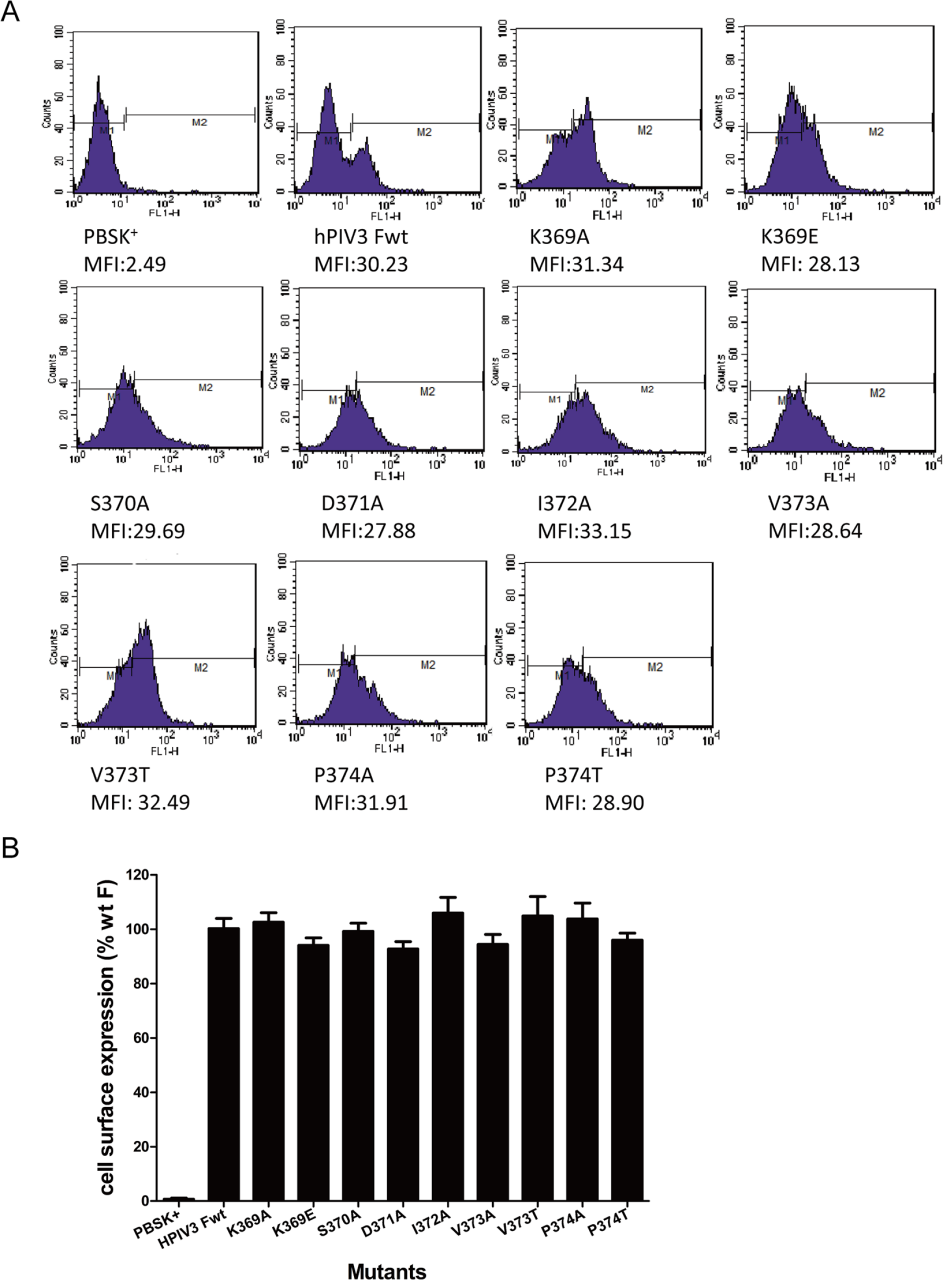
Cell surface expression (CSE) of the mutant F proteins. Cell surface expression was measured by flow cytometry with an anti-F monoclonal antibody followed with an anti-mouse Alexa Fluor 488-conjugated antibody. (A) Representative fluorescent histograms for each mutant were shown. The x axis indicates the fluorescent intensity values shown in log scale and the y axis represents cell counts, respectively. The mean fluorescence intensity (MFI) of each mutant was calculated and listed below. (B) Quantitation of the cell surface expression level. The mean fluorescence intensity of cells expressing the mutated F proteins was represented as a percentage of that of the wt F subtracting background labeling of control cells transfected with the vector alone. The means and standard errors are from triplicate experiments.

To investigate whether the lack of fusion activity of these mutated F proteins was ascribed to their reduced levels of cell surface expression, fluorescence-activated cell sorting (FACS) analysis was executed with an anti-F monoclonal antibody as described in Materials and Methods to examine the relative cell surface expression levels of these mutated F proteins. Expression level was quantified by the mean fluorescence intensity per cell and expressed as a percentage of that of the wt F subtracting background labeling of control cells transfected with the vector alone. Fluorescent histograms of the wt or mutated F proteins were shown in Fig 6A. Quantitation of the cell surface expression, expressed as a percentage of the wt F protein level, are summarized in Fig 6B. Each of the mutated proteins gave a mean fluorescent intensity that was similar to that of the wt F protein, ranging from 93.1% to 105.9% of the wt F level. Therefore, the loss of fusion activity was not due to the diminished cell surface expression level.

### Mutations in the DI-DII linker exhibited defects in the F-HN interaction

**Fig 7:**
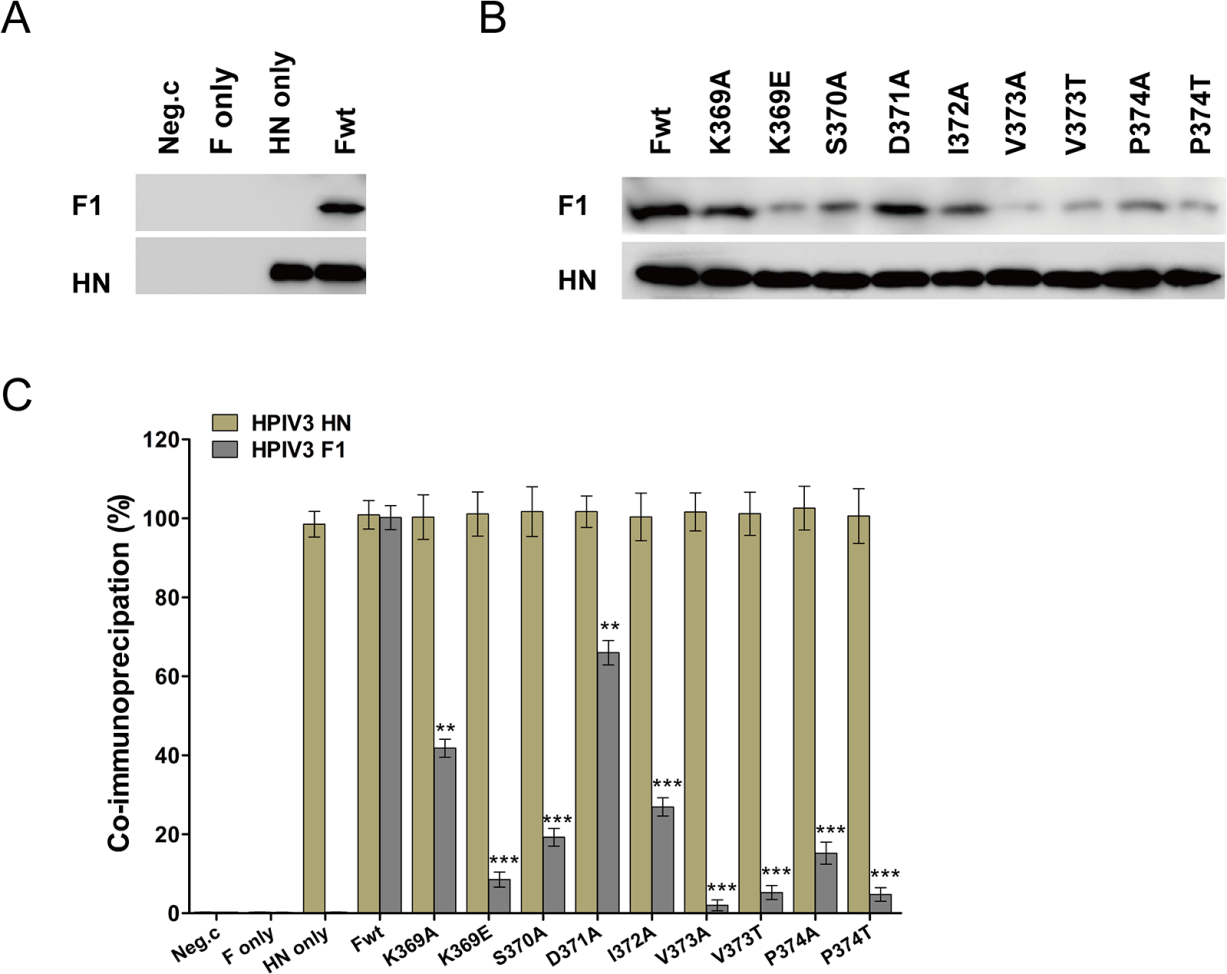
Co-immunoprecipitation analyses of the interactions of the wt or mutated F with HPIV3HN proteins. The surface proteins of the transfected cells were biotinylated, then the cells were lysed and the lysates were incubated with Dynabeads-cross-linked anti-HN monoclonal antibodies. After forming Dynabeads-HN Ab-HN Ag-F Ag immune complexes, they were solubilized in PBS and then incubated with streptavidin beads at 4°C all night to precipitate the cell surface complexes. Precipitated complexes were separated on a PVDF membrane and probed with anti-F monoclonal antibody (top panels) or a mixture of two anti-HN monoclonal antibodies (bottom panels). All proteins were electrophoresed in the presence of reducing agent. (A) The critical controls of this co-IP assay: Neg.c, empty vector; HN only and F only, these plasmids alone; wild-type F protein (Fwt) and HN. (B) The results of a co-immunopreciptation assay of HPIV3 F proteins carrying mutations in the DI-DII linker. Wild-type F protein (Fwt) and the nine mutants cotranfected with HN are indicated above the gels. (C) The quantitative results for Fig 7B, using densitometry method from three independent experiments. The relative levels of the co-immunoprecipitated F proteins for the mutants are expressed as the percentages of that detected in cells transfected with wt F and HN genes. Error bars indicate the standard deviations (**P<0.01, ***P<0.001).

To further ascertain if the deficiencies in fusogenic activity of these mutated F proteins were owing to interfering with formation of the HN-F complex at the cell surface, a co-immunoprecipitation assay was done with a mixture of two anti-HN protein monoclonal antibodies to test for the ability of each mutated F protein to interact with HPIV3 HN at the surface of HN-F-cotransfected cells. For this assay, the F protein in the immunoprecipitates was detected by Western blotting analysis using an anti-F monoclonal antibody, while the HN protein was detected in a separate Western blotting using two anti-HN monoclonal antibodies. Fig 7A shows critical controls. The first lane showed that neither protein is detected in cells transfected with vector alone. The second lane demonstrated that the F protein is not co-immunoprecipitated by a mixture of anti-HN monoclonal antibody in the absence of the HN protein. The third lane demonstrated that the F protein that is co-immunoprecipitated with HN is not present in cells that have not been tranfected with the F gene. These controls established the specificity of the co-IP of F by the anti-HN antibodies. The relative levels of F proteins that co-immunoprecipitated from extracts of cells cotransfected with mutant F and HN genes were expressed as a percentage of that co-immunoprecipitated from cells coexpressing wt F as well as its homologous HN proteins. Fig 7B showed the co-immunoprecipitation results that all of the mutated F proteins reserved the ability to co-IP with HPIV3 HN. The quantification data are summarized in Fig 7C, the K369E, V373A, V373T and P374T mutated F proteins in the presence of HPIV3 HN coexpression, each of which fused less than 19% of the wt level, almost lost their abilities to co-IP with HN protein, corresponding to percentages of less than 10.0%. The S370A mutant both fused and co-immunoprecipitated at approximately one fifth of the wt F level. Moreover, I372A- and P374A- mutated F, which fused at 57.5% and 54.5% of the wt level, co-IP at 26.9% and 15.2%, respectively. K369A and D371A mutated F proteins, which had fusion activity at 71.3% and 69.4% of the wt level, co-immunoprecipitated at 41.4% and 66.0%, respectively. These findings demonstrate that the reduction in the ability of each mutated F protein to interact with their homologous HN protein at the cell surface may be responsible for the loss of fusion activity.

## Discussion

Despite mutational analysis of the fusion peptide [34–36], HRA [37, 38], HRB and its linker [39–41], and domains DI, DII [42] of the paramyxoviruses F proteins have found that these regions had important effects on F-mediated membrane fusion, the possible role of the DI-DII linker of the HPIV3 F protein in cell-cell fusion still remains to be elucidated. To study the role of this domain in fusion activity of F protein, the prefusion HPIV3 F protein structural model was generated to identify the new location of this linker in it, and site-directed mutagenesis was utilized to replace each amino acid in this domain to form nine single mutations. Cell surface expression and F-HN interaction, which may affect the fusogenicity of F, were also analyzed. The results showed that amino acid substitutions in this domain which still located between DI and DII in the prefusogenic state of HPIV3 F resulted in mutant proteins defective in fusion activity in all stages of the fusion process.

The influences of these mutants in the DI-DII linker on fusion activity of HPIV3 F protein were determined by using three different types of membrane fusion assays, which corresponds to the three sequential stages in the process of membrane fusion. Both the dye transfer assay and the kinetics of fluorescence dequenching assay were used to measure the merger of the outer leaflet membrane lipids of the donor and recipient cells [43, 44]. The content mixing assay was employed to quantify the mixing of cytoplasms of fusing cells [38, 45]. The syncytium formation assay was conducted to analyze the formation of multinucleated giant cells which is the result of pore expansion in the final stage of the membrane fusion [35, 38]. Intriguingly, although all of the amino acid substitutions in the DI-DII linker of HPIV3 F protein exhibited decreased fusogenicity, not all residues were of equal effects on F-induced cell-to-cell fusion, since five mutations (i.e., K369E, S370A, V373A, V373T and P374T) exhibited fusion activity less than 19%, and the remaining mutants (i.e., K369A, D371A, I372A, P374A) in this domain retained this activity slightly more than half of that of wt F level. Further analysis of the five mutants which almost eliminated their abilities to form syncytia and drastically weakened their content mixing abilities, with the rate and extent of initial fusion simultaneously diminished more than 85% of those of the levels of wt F suggested that the very slow fusion kinetics of them unable to efficiently support pore enlargement may result in the loss of syncytium formation abilities.

The expression efficiency of each mutated F protein on the cell surface was similar to that of the wt F protein, indicating that these mutants retained the ability to be folded correctly and be transported efficiently to the cell surface, since it is generally held that due to a cellular quality control system, only the properly structured and assembled viral envelope proteins could transport through the secretory pathway to the cell surface, and the proteins that are misfolded or assembled aberrantly are usually recognized and retained in the endoplasmic reticulum (ER) [46]. Besides, it can be revealed from the co-IP assay that the migrations of all mutated F proteins on polyacrylamide gels in the presence of reducing agent were roughly comparable to that of the wild-type protein, implying that the oligosaccharide processing was normal and the molecular weights of the mutants and wt F proteins were very similar. Taken together, these data suggested that the structures of the mutated proteins may keep intact and the diminished fusion activity could not be accounted for by any obvious defect in cell surface expression or a gross conformational difference.

As is typical of many paramyxoviruses, a virus-specific interaction between F and HN proteins is crucial and required for fusion activation [47, 48]. Thus, a co-immunoprecipitation assay was carried out to detect the specific HN-F interaction at the surface of cells coexpressing HPIV3 wt or mutated F and HN proteins. The results showed that all of the mutated F proteins were not only deficient in fusion activity in the presence of HPIV3 HN coexpression but also led to a decrease in the amount of F that co-IPs with HN. These findings coincide with previous results demonstrating that the defective fusion activity correlates with a decreased amount of functional complexes containing the HPIV3 F and HN proteins [49]. Therefore, one plausible hypothesis for the deficiency in fusion activity of these mutated F proteins may be that they were defective in interacting with HPIV3 HN at the cell surface, while this interaction could allow HN protein receptor binding to stimulate the drastic conformational changes in F protein to expose the hydrophobic fusion peptide at the right time and in the right place into the target membrane to induce membrane fusion. Indeed, we can not exclude the possibility that the DI-DII linker per se was part of its HN protein-interacting domain, since this region corresponded to the P2 region of the PIV2 F protein which was responsible for the specificity of the PIV2 F protein for PIV2 HN protein [50].

It has been reported that the DI-DII linker interacted with FP in the prefusion form of the PIV5 F protein [51], and in our newly generated HPIV3 F 3D model in the prefusion form, residues 369–374 still act as the DI-DII linker, so another possible hypothesis for the defective fusion activity of the HPIV3 F protein is that the mutations in the DI-DII linker interfere with its interaction with the FP, and then disturb a conformational change induced by the HN protein that is required for membrane fusion.

In summary, the substitutions we have introduced into the DI-DII linker of the HPIV3 F glycoprotein exhibited defective fusogenicity in all steps of the membrane fusion process, suggesting that this region has an important effect on fusion activity. Deeper understanding of the role of different regions of HPIV3 fusion protein in regulating the membrane fusion activity will facilitate the development of safe and effective vaccine strategies.
